# P37 Significance of a swollen eye in a child with Down syndrome

**DOI:** 10.1093/rap/rkab068.036

**Published:** 2021-10-19

**Authors:** Charlene Foley, Louise Allen, Peter Bale

**Affiliations:** Addenbrooke's Hospital, Cambridge, United Kingdom

## Abstract

**Case report - Introduction:**

Due to dysregulation of their immune system, children with Down syndrome are at increased risk of a number of conditions, including malignancy, infection and autoimmune disorders. This case highlights the importance of the MDT team working in order to fully explore possible diagnoses and reach the correct conclusion. We present a case of a relatively rare eye disorder, initially treated as peri-orbital cellulitis, that required a collaborative team approach to reach the final diagnosis.

**Case report - Case description:**

A 6-year-old-girl with Down syndrome was referred to the oncology and tertiary ophthalmology teams by her local paediatric team with a history of recurrent right-eye peri-orbital swelling. The impression had been of recurrent peri-orbital cellulitis which was treated with and responsive to antibiotics. However, the most recent presentation was unresponsive to antibiotics and had associated orbital symptoms of proptosis and restricted eye movement.

An MRI-head showed a protruding right eye with an enhancing mass centred in the right-superior temporal-extraconal-orbit. The mass was infiltrating the extra-ocular muscles and there was low bone-marrow signal compared to the left-side. The MRI findings were discussed in the oncology MDT-meeting. Appearances were felt to be more in keeping with malignancy, rather than infection. An ophthalmology review confirmed no evidence of optic-nerve compression, so she was discharged on antibiotics with a plan made to admit for biopsy and bone marrow aspirate under general anaesthetic.

Histology of the lesion was reported to show a mixed inflammatory-mass, angiocentric with no elements of neoplasia. Oncology-specific stains were negative. BMA was normal. The differential at this point was orbital-pseudo-tumour; if infection and systemic-auto-immune conditions could be excluded. Histochemical-staining of the biopsy excluded fungi, bacteria or mycobacteria. The granulomatous-nature of the infiltrate and the conspicuous presence of eosinophils were consistent with an autoimmune-condition. The little girl was discharged from the oncology team into the care of the ophthalmology and paediatric rheumatology teams for further work-up and management. She was commenced on prednisolone.

On rheumatology-review, focussed-history and examination were unremarkable in terms of an underlying systemic-inflammatory condition. A full-blood-work-up was requested. Her pANCA and MPO were mildly raised, as were her inflammatory markers. In view of these results, a CT chest/abdomen angiogram was arranged which was normal. A diagnosis of orbital-pseudotumour was made. There was clinical improvement on prednisolone, so a tapering plan was provided.

**Case report - Discussion:**

First described by Birch-Hirschfield in 1905, orbital pseudo-tumour, also known as idiopathic orbital inflammatory syndrome (IOIS) is a benign, non-infective, inflammatory condition of the orbit without identifiable local or systemic causes. Post-treatment recurrence occurs in 37% of cases.

In this case, as steroids were weaned, there was recurrence of eye symptoms with proptosis and redness of the eye. Repeat bloods were performed, and an MRI-head requested. The repeat ANCA-MPO was negative. Whilst awaiting her MRI, a further clinical review was carried out jointly by paediatric rheumatology and ophthalmology. At this appointment, there was notable proptosis which appeared to be getting worse. The impression was that there was recurrence of the inflammatory swelling, and that the sensible option would be to restart treatment with oral prednisolone. At this point, addition of a steroid-sparing DMARD was discussed. Methotrexate was chosen and commenced.

The little girl continued to attend for joint rheumatology and ophthalmology reviews. She continued to have proptosis in the right eye but had excellent ocular motility and good visual-acuity. She was noted to develop a worsening in the lid swelling and possible proptosis with an intercurrent illness, perhaps suggesting that the underlying inflammatory process was still active. The plan was to continue methotrexate for 2 years and perform another orbital MRI before potentially reducing methotrexate. However, in this time the methotrexate dose was reduced due to deranged LFTs.

She continued to have “flare ups” with increasing proptosis despite treatment with methotrexate. A repeat MRI after 2 years of treatment showed reduction in the size of the right supra-lateral orbital mass compared to the first scan. However, there was uniform enhancement following gadolinium injection with mild proptosis of the right-globe. The appearances were in keeping with an active granulomatous lesion.

The current plan is to try and re-optimise the dose of methotrexate. If not tolerated, an alternative DMARD will need to be considered.

**Case report - Key learning points:**

The diagnosis of orbital pseudo-tumour is one of exclusion with evaluation directed to exclude neoplasms, infections, and systemic inflammatory disorders. It can manifest as a bilateral condition in 13% and with constitutional symptoms in 40%.

**Points for discussion:**

- Differential of orbital pseudo-tumours in the paediatric population
- Prognosis of pseudo-tumours
- Suggested treatment pathway -- Is control to allow normal eye movement enough or should we be treating more aggressively?

